# Quantification of baseline amyloid PET in individuals with subjective cognitive decline can identify risk of amyloid accumulation and cognitive worsening: the FACEHBI study

**DOI:** 10.1007/s00259-025-07270-7

**Published:** 2025-04-23

**Authors:** Guilherme Domingues Kolinger, Oscar Sotolongo-Grau, Núria Roé-Vellvé, Juan Pablo Tartari, Ángela Sanabria, Esther Pérez-Martínez, Norman Koglin, Andrew W. Stephens, Montserrat Alegret, Lluís Tárraga, Miren Jone Gurruchaga, Agustín Ruiz, Mercè Boada, Santiago Bullich, Marta Marquié, on behalf of the FACEHBI study group, N. Aguilera, E. Alarcón-Martín, M. Alegret, J. A. Alllué, P. Bayón-Bujan, N. Bein, M. Berthier, J. Blázquez-Folch, M. Boada, M. Buendia, S. Bullich, F. Campos, B. Calm-Salvans, A. Cano, F. Casales, P. Cañabate, L. Cañada, C. Cuevas, I. de Rojas, S. Diego, G. Domingues-Kolinger, J. M. Escudero, A. Espinosa, M. V. Fernández, A. Gailhajenet, P. García-González, J. Giménez, M. Gómez-Chiari, M. Guitart, P. C. Gutiérrez-Rodríguez, I. Hernández, M. Ibarria, A. Lafuente, F. Lomeña, M. Marquié, E. Martín, M. Moreno, A. Morera, L. Montrreal, N. Muñoz, A. Muñoz-Morales, A. Niñerola, A. B. Nogales, L. Núñez, C. Olivé, A. Orellana, G. Ortega, A. Páez, A. Pancho, E. Pelejà, E. Pérez-Martínez, A. Pérez-Cordon, V. Pérez-Grijalba, M. Pascual-Lucas, A. Perissinotti, S. Preckler, R. Puerta, V. Pytel, M. I. Ramis, J. Rodríguez, N. Roé-Vellvé, J. Romero, M. Rosende-Roca, A. Ruiz, A. Sanabria, P. Sanz-Cartagena, M. Sarasa, S. Seguer, O. Sotolongo-Grau, A. Stephens, J. P. Tartari, L. Tárraga, M. A. Tejero, J. Terencio, M. Torres, S. Valero, L. Vargas, A. Vivas, on behalf of the AMYPAD consortium, Frederik Barkhof

**Affiliations:** 1 Life Molecular Imaging GmbH, Berlin, Germany; 2 Ace Alzheimer Center Barcelona – Universitat Internacional de Catalunya, Barcelona, Spain; 3 Centro de Investigación Biomédica en Red de Enfermedades Neurodegenerativas (CIBERNED), Instituto de Salud Carlos III, Madrid, Spain; 4 Glenn Biggs Institute for Alzheimer’s & Neurodegenerative Diseases, University of Texas Health Science Center at San Antonio, San Antonio, TX USA

**Keywords:** Amyloid PET, Subjective Cognitive Decline, Alzheimer’s disease, Florbetaben, Longitudinal study, FACEHBI

## Abstract

**Purpose:**

Amyloid PET imaging is capable of measuring brain amyloid load in vivo. The aim of this study is to assess the relationship of the baseline amyloid with its accumulation over time and with cognition in individuals with subjective cognitive decline (SCD), giving a focus on those below Aβ positivity thresholds.

**Methods:**

118 of 197 individuals with SCD from the Fundació ACE Healthy Brain Initiative underwent three [^18^F]florbetaben scans and the remaining 79 underwent two scans in a 5-year span. Individuals were categorised based on baseline Centiloid values (CL) into amyloid positive (Aβ+; CL > 35.7), Grey Zone (GZ; 20 < CL ≤ 35.7), and amyloid negative (Aβ-; CL ≤ 20). Relationship between conversion to mild cognitive decline (MCI) and baseline amyloid levels was assessed. Then, to focus on sub-threshold individuals with amyloid accumulation, the Aβ- group was split into two groups (N1 (CL ≤ 13.5) and N2 (13.5 < CL ≤ 20)), Aβ accumulation was determined, and a parametric image analysis of the Aβ accumulators in the N1 group was performed.

**Results:**

At baseline, 20 individuals were Aβ+, 8 GZ, 160 N1, and 9 N2. Higher Aβ load, older and less educated individuals presented increased risk of MCI-conversion. Longitudinally, 19% of N1 individuals were accumulators despite very low Aβ burden at baseline. Meanwhile, 89% of the N2 group accumulated Aβ as well as all GZ individuals (which had the highest rate of amyloid accumulation, 5.1 CL/year). In the parametric image analysis of N1 accumulators, a region within the precuneus was linked to increased Aβ over time.

**Conclusion:**

Baseline amyloid levels differentiate individuals who accumulate amyloid over time and that are at risk for cognitive decline, including those at sub-threshold levels of Aβ. This can be valuable to identify pre-clinical AD in a SCD population.

## Introduction

With the recently developed disease modifying drugs for Alzheimer’s disease (AD), it becomes important to establish an accurate diagnosis in the early stages of the disease continuum and to track disease progression through clinical features and biomarkers so patients can be enrolled and followed-up appropriately [1–8]. Therefore, studying individuals with subjective cognitive decline (SCD) is key to understand the earliest stages of AD. SCD refers to the self-perception of cognitive decline that is not yet captured on conventional standardized cognitive tests [9]. Compared to the general population, individuals with SCD have a higher risk of developing AD dementia, especially if they present specific clinical and biomarker features [9]. Therefore, the SCD population provides an early window to study and understand AD before the brain is irreversibly damaged by the disease natural trajectory.

One of the main and earliest hallmarks of AD pathology is the aggregation of the amyloid-beta (Aβ) protein in the brain [10–12], which can be detected in-vivo with positron emission tomography (PET) [13–15]. Aβ PET imaging has been investigated for the early detection of Aβ deposition, since Aβ plaques can be detected two decades before AD clinical symptoms begin [2, 12]. One of the main strengths of Aβ PET comes from the direct detection and measurement of amyloid burden through reliable image quantification. This allows also for an early detection of Aβ burden, increased inter-reader agreement, increased image read confidence, and monitoring of disease progression and plaque clearance upon anti-amyloid therapy [16–22]. In fact, these merits of Aβ PET quantification have made it a staple of AD research and clinical trials of disease modifying drugs [1, 8, 23, 24]. Individuals with SCD have been assessed not only with Aβ PET imaging but also with other imaging modalities and with fluid biomarkers [16, 25–33], which showed good correlation with PET and cognitive decline. A more detailed assessment of the SCD population with Aβ levels below pathological threshold is warranted and could help address open questions regarding the brain amyloid accumulation trajectory and distribution pattern in this sub-threshold group. For instance, it is interesting to understand if individuals in this low Aβ group who eventually exhibit Aβ accumulation show a distinct baseline brain Aβ PET pattern compared to those with stable Aβ levels. Moreover, if such pattern exists and aligns with previous literature findings of Aβ accumulation in SCD populations, that will further support existing evidence on early brain changes associated with amyloid pathology.

The present study aimed to quantify the amyloid deposition in individuals with SCD from the Fundació ACE Healthy Brain Initiative (FACEHBI) and to follow their Aβ and cognitive status over time. An analysis focusing on individuals with very low levels of amyloid at baseline but with measurable Aβ yearly accumulation was performed to differentiate these individuals from those Aβ- without Aβ increase and to identify regional patterns of early amyloid load.

## Materials and methods

### Participants

FACEHBI is a longitudinal study of aging, cognition, lifestyle and biomarkers in individuals with self-reported SCD performed at Ace Alzheimer Center Barcelona (Ace) in Barcelona, Spain, focused on the analysis of clinical, neuroimaging and molecular features relevant in pre-clinical AD [34].

Some key inclusion criteria were: age above 49 years old, Mini-Mental State Examination (MMSE [35, 36]) score ≥ 27 and Clinical Dementia Rating (CDR [37]) = 0. Further details on inclusion and exclusion criteria can be found on previous publications [28, 32, 34]. Of note, the FACEHBI population does *not* fit in the SCD plus classification [9] and represents an even earlier stage of possible pre-clinical AD. The main AD-related biomarker in FACEHBI is amyloid PET imaging with [^18^F]florbetaben, which was performed at baseline and longitudinally every two or three years. The present work included FACEHBI participants that underwent at least two [^18^F]florbetaben PET scans. Therefore, the FACEHBI cohort represents one of the best characterised, longitudinally followed cohorts of early sporadic Alzheimer’s disease.

A written consent was obtained from all participants prior to the enrolment in the study. The FACEHBI protocol received approval from the ethics committee of Hospital Clínic i Provincial in Barcelona, Spain (EudraCT number 2014–00079 - 38). The referral centre ethics committee approved the patient recruitment, and collection protocols were in accordance with ethical standards according to World Medical Association Declaration of Helsinki—Ethical Principles for Medical Research Involving Human Subjects.

### Longitudinal design

**Fig. 1:**
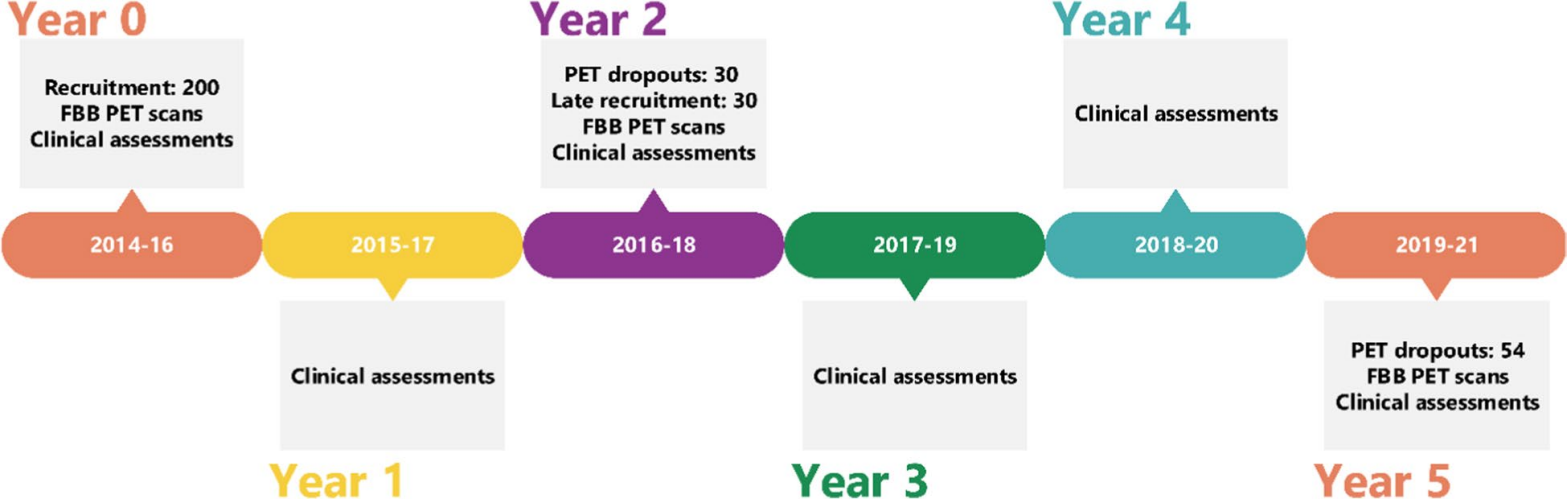
FACEHBI study timeline

Participants have been followed yearly for up to 5 years after the initial visit. Neurological and cognitive assessments were carried out yearly while amyloid PET scans, brain magnetic resonance imaging (MRI), and other biomarkers were performed on the initial visit (Year 0) and on Years 2 and 5 (Fig. 1). To address dropouts between the first and second PET scan (n = 30), 30 participants with SCD were recruited in Year 2. Thus, the *baseline visit* of these subjects was on Year 2 and they had a shorter follow-up time. Additionally, a total of 54 individuals did not undergo PET scanning on Year 5, either dropping out after Year 0 and Year 2 participation (n = 51) or late-enrolment individuals who decided not to have a follow-up (n = 3).

### Cognitive evaluation

Participants underwent an extensive neuropsychological protocol that included the neuropsychological battery of Fundació ACE (NBACE) [38, 39], the Spanish version of the face-name associative memory exam (S-FNAME) [40, 41], and a set of self-administered questionnaires [34]. All participants had a diagnosis of SCD at baseline and their neurological and cognitive status was reevaluated yearly, assessing their potential conversion to mild cognitive impairment (MCI). A diagnosis of MCI was endorsed if performance in any of the NBACE test scores was impaired according to the published cutoffs [38, 39] and participants maintained their autonomy in daily activities.

### Image acquisition

#### Magnetic resonance imaging

MRI scans were acquired prior to PET scans in a 1.5 T Siemens Magneton Aera (Erlangen, Germany) using a 32-channel head coil at the *Clínica Corachan*, Barcelona. Anatomical T1-weighted images were acquired using a rapid acquisition gradient-echo 3D MPRAGE sequence with the following parameters: TR 2.200 ms, TE 2.66 ms, TI 900 ms, slip angle 8º, FOV 250 mm, slice thickness 1 mm and isotropic voxels of 1 × 1 × 1 mm [34]. Other MRI sequences were also acquired for the FACEHBI study but were not used in the current work.

#### [^18^F]Florbetaben amyloid PET

[^18^F]florbetaben PET/CT scans were acquired in a Siemens Biograph mCT scanner at the Radiology Department from the *Hospital Clínic i Provincial* in Barcelona. Twenty-minute PET scans were acquired 90 min after the injection of 300 MBq [^18^F]florbetaben [42].

### Image processing & quantification

Frames from the amyloid PET images were motion corrected, and an average was calculated to generate a static single frame PET image. Quantification of the amyloid scans was performed in MNI space and followed the Centiloid (CL) method [18, 19]. Additionally, quantification was also performed in target regions defined by the Automated Anatomical Labelling (AAL) atlas [43] masked by the grey matter segmented from each individual’s T1-MRI. Whole cerebellum was used as reference region in all cases. Data processing and quantification was performed using MATLAB, Statistical Parametric Mapping version 8 (SPM8) and in-house quantitative tools based on SPM8.

### Data analysis

#### Centiloid classification

**Fig. 2:**
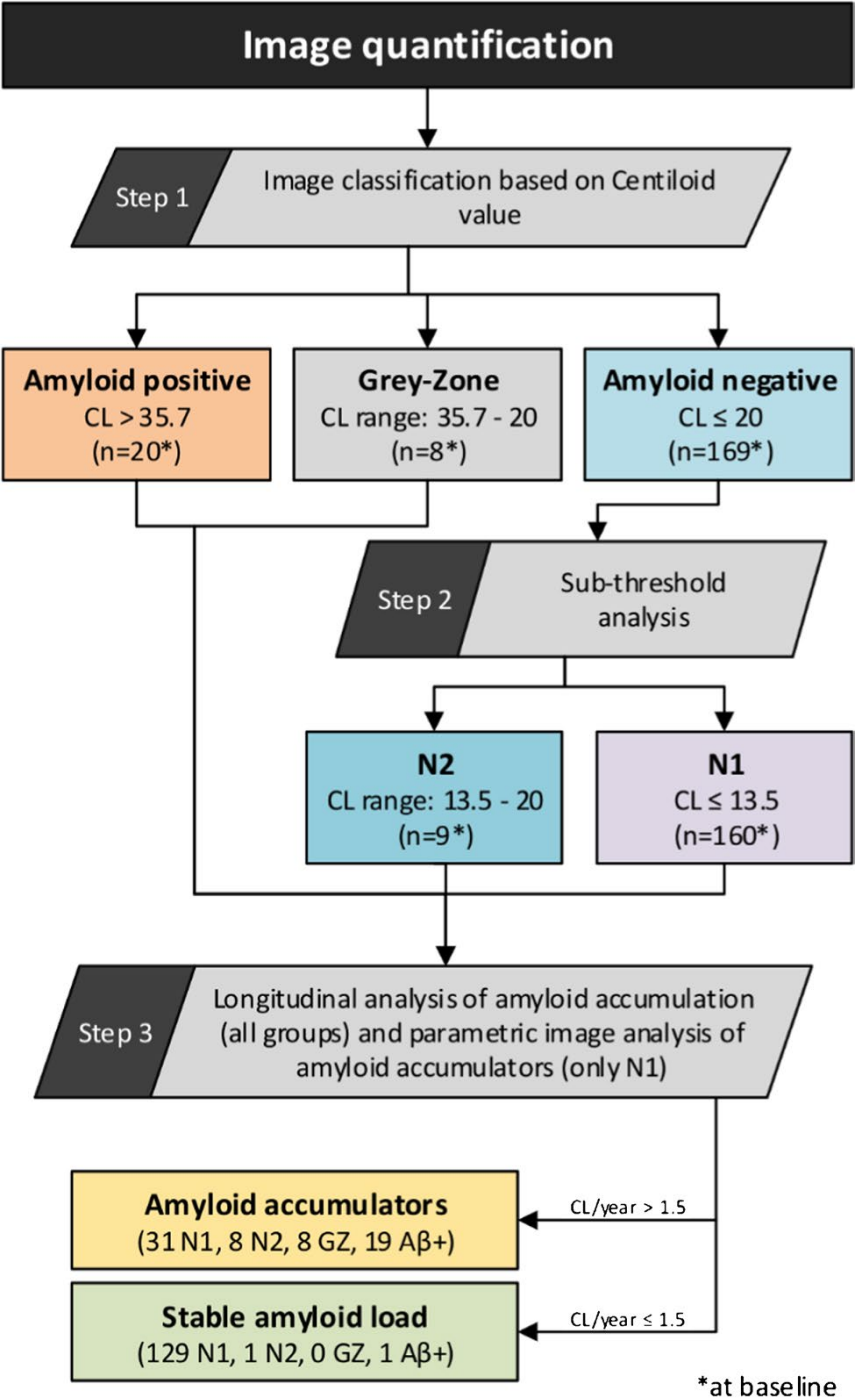
Centiloid classification, split into groups, and the sequential steps of the analyses carried in this study. Number of individuals in each group at baseline is indicated with an asterisk

As a first step, amyloid-beta positivity (Aβ+) was defined at CL > 35.7 (via a histopathology cohort), negativity (Aβ-) was defined at CL ≤ 20 (via a healthy elderly cohort), and the intermediary range between 20 < CL ≤ 35.7 was defined as a *“Grey Zone”* (GZ) [2, 17]. Then, to study very early stages of amyloid deposition, the Aβ- range was further split into N1 (CL ≤ 13.5) and N2 (13.5 < CL ≤ 20) based on an early Aβ detection cut-off [2]. Finally, individuals were classified either as Aβ accumulators or with stable Aβ load (Fig. 2).

#### Risk of cognitive decline

A Cox Proportional Hazard model established a relationship between the baseline CL and subsequent risk of cognitive decline to MCI while considering relevant covariates. For that, the conversion to MCI was considered as event and individuals were grouped based on their baseline CL (Aβ- *vs* GZ *vs* Aβ+). First, baseline age, years of education, MMSE, sex and APOE-ε4 genotyping were compared between the stable SCD and MCI-converter groups. Only those variables that were significantly different between groups were included as covariates in the final model. Individuals were considered to have converted to MCI if their latest clinical assessment showed cognitive impairment on the NBACE while maintaining autonomy in their daily activities, and for those participants the date of conversion was their first assessment as MCI.

#### Centiloid Annualised Rate of Change (ARC)

A single linear mixed effects model (LMM) was used to estimate the CL Annualised Rate of Change (ARC; a CL/year accumulation rate) for each subject and CL group as described above (Aβ+, GZ, N2, or N1). This model was configured with CL value as a function of the time from baseline scan and the CL baseline group (categorical variable) as fixed effects, and time from baseline as random effect. As such, intercepts and slopes for CL groups (defined at baseline) and for each individual were derived. Intercepts represent a model estimate for baseline CL and slopes are the CL accumulation rate. To establish if an individual has substantial amyloid accumulation, a Gaussian Mixed Model (GMM) with two curves was applied to the CL ARC distribution of the whole cohort. Individuals with an ARC above the mean plus 3 times the standard deviation of the lowest curve of the GMM model were considered Aβ accumulators (1.5 CL/year; see Results).

#### N1 spatial pattern parametric analysis

To assess amyloid accumulation and early signal in a sub-threshold population, parametric SUVR images of the N1 group were calculated and the spatial pattern of the baseline scans of Aβ accumulators was compared against those with stable Aβ. This comparison was performed with SPM voxel-wise SUVR analysis using an unpaired t-test at significance level of p < 0.001 for clusters of at least 125 voxels (1 mL).

## Results

### Baseline amyloid PET scans

**Fig. 3:**
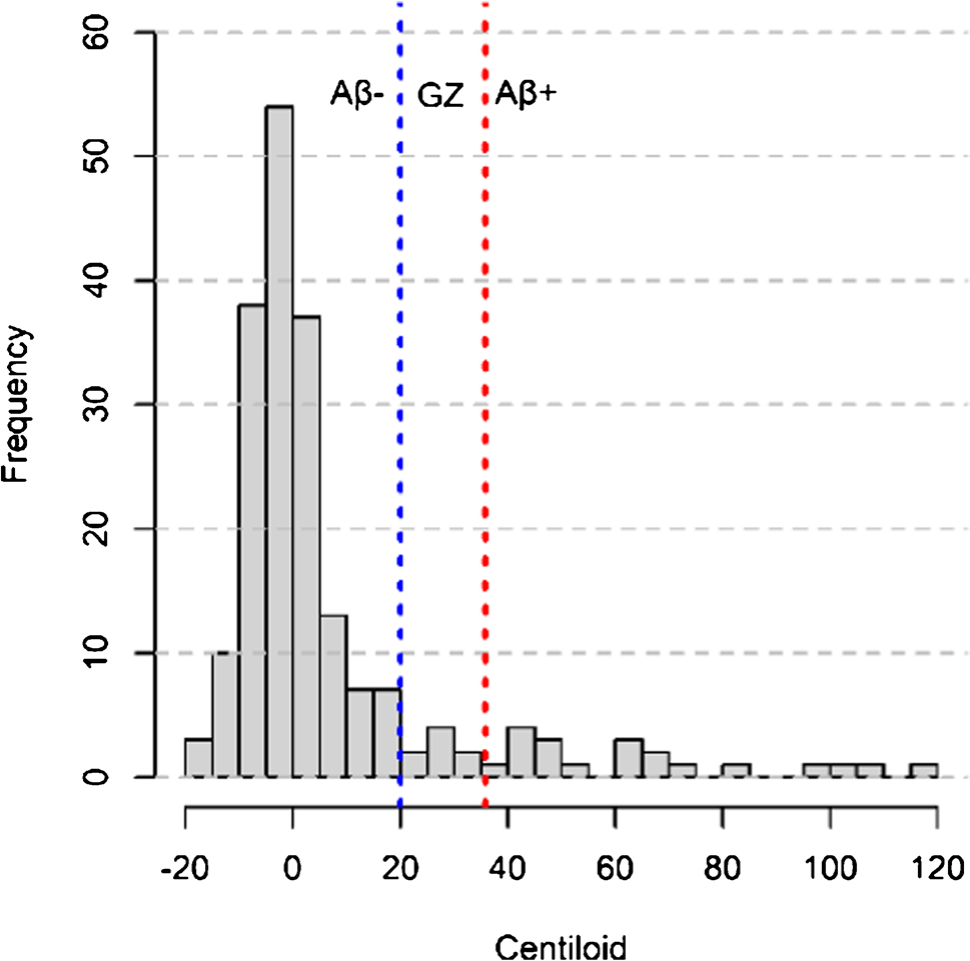
Histogram of Centiloid values at baseline. Vertical lines display the cut-offs for Aβ- (CL ≤ 20, blue) and Aβ+ (CL > 35.7 red)

In total, 197 participants (F/M = 125/72) with an average age of 65.5 ± 7.2 years, 15.5 ± 4.5 years of education and an MMSE of 29.3 ± 0.9 at baseline were included in the analysis (Table 1). Most participants were classified as Aβ- (n = 169/197 (86%), CL ≤ 20), but several individuals presented abnormal amyloid deposition (n = 20/197 (10%), CL > 35.7) and some showed emerging amyloid deposition (n = 8/197 (4%), 20 < CL ≤ 35.7). The histogram of baseline CL is displayed in Fig. 3.

**Table 1:** Demographics, PET assessment, and cognitive scores of individuals at baseline

	Aβ- (CL ≤ 20)	Grey Zone (20 < CL ≤ 35.7)	Aβ+ (CL > 35.7)
*N*	169	8	20
Female:Male	111:58	4:4	10:10
Age*	64.7 ± 7.3	69.8 ± 3.7	69.8 ± 4.8
Baseline CL	− 1.0 ± 7.2	28.0 ± 3.8	65.3 ± 24.3
APOE-ε4 carriers*	34	3	13
Years of education	15.4 ± 4.4	14.1 ± 5.5	14.3 ± 4.9
MMSE	29.3 ± 0.9	28.9 ± 0.8	29.6 ± 0.8
Aβ visual read FBB scan (negative:positive)	168:1	5:3	5:15

*Aβ- group is significantly younger than Grey Zone and Aβ+ individuals (Welch ANOVA with Games-Howell post-hoc test, *p* ≤ 0.01) and APOE-ε4 expression differs per group (Pearson’s chi-squared test with simulated *p*-value, *p* ≤ 0.01). Years of education and MMSE are not different between CL groups (Kruskal–Wallis rank sum test, *p* > 0.05)

### Risk of progression to MCI

All individuals had a diagnosis of SCD at their initial clinical evaluation. According to follow-up assessments, the cohort was divided into stable-SCD (n = 151/197) (individuals with a SCD diagnosis at their last clinical evaluation), and MCI-converters (n = 46/197) groups (individuals that showed cognitive decline and had an MCI diagnosis at their latest follow-up point). Follow-up time, baseline demographic, cognitive and PET characteristics of both groups are compared in Table 2. MCI-converters showed significantly higher CL, were older and less educated.

**Table 2:** Features of the stable SCD and MCI converter groups

Covariate	Stable SCD (*n* = 151)	MCI converters (*n* = 46)	Test and *p*-value
Follow-up time	54.9 ± 13.0 months	57.8 ± 11.9 months	Wilcoxon rank sum *p* = 0.06
Baseline Centiloid	3.37 ± 17.5 CL	18.47 ± 32.8 CL	Wilcoxon rank sum ***p* = 0.01**
Age	64.2 ± 7.0 years old	69.7 ± 6.1 years old	Wilcoxon rank sum ***p* < 10**^**–5**^
Sex	98F / 53M	27F / 19M	Chi-squared *p* = 0.56
Years of education	15.7 ± 4.2 years	13.6 ± 5.0 years	Wilcoxon rank sum ***p* < 0.01**
APOE-ε4 carriers	118 non-carriers 33 carriers	29 non-carriers 17 carriers	Chi-squared *p* = 0.06
MMSE	29.3 ± 0.96	29.2 ± 0.79	Wilcoxon rank sum *p* = 0.43

Values are presented as mean and standard deviation for relevant metrics. Statistical tests and resulting *p*-values are presented. Significant p-values (*p* < 0.05) are in bold text

#### Baseline amyloid PET and MCI-conversion

**Fig. 4:**
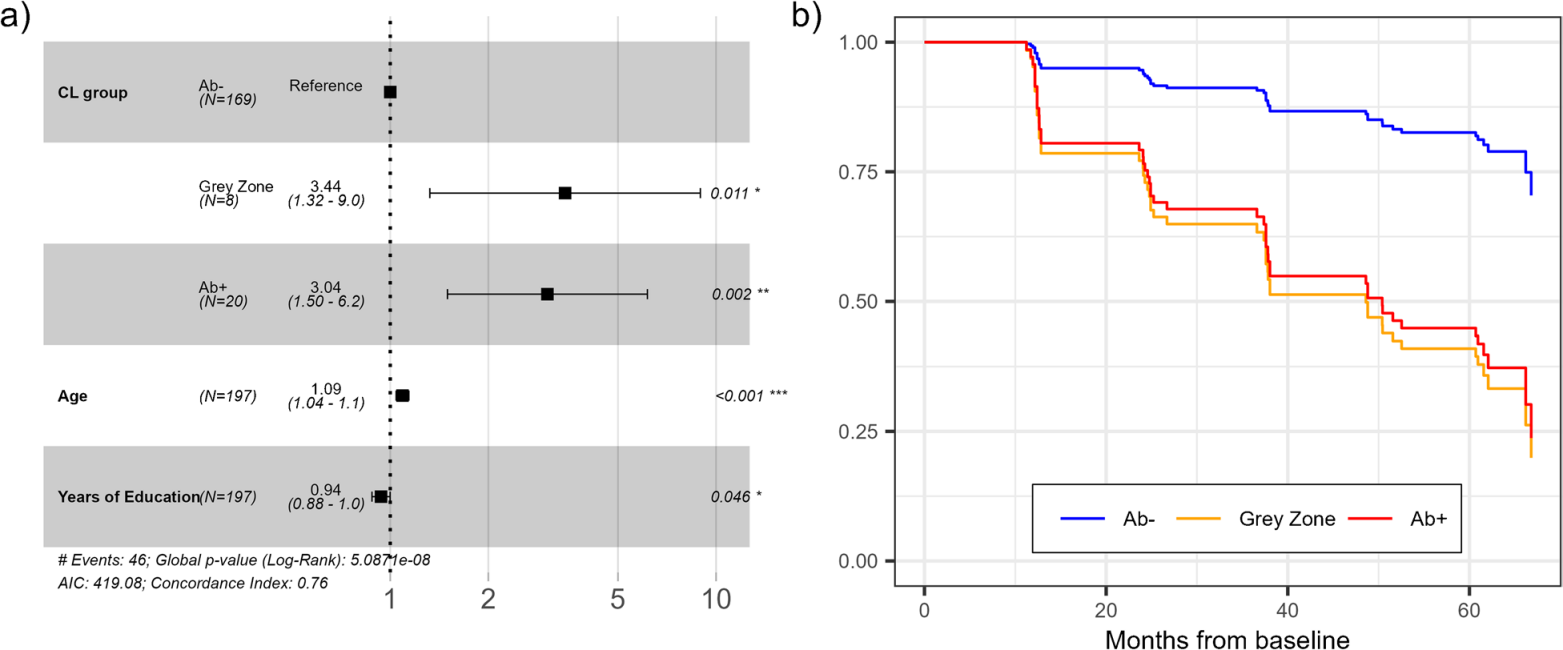
**A** Cox Proportional Hazard analysis of the risk of SCD to MCI conversion based on the CL level at baseline, age, and education. **B** Kaplan-Meyer-like adjusted curves indicating the likelihood of remaining SCD over time

Of the 169 Aβ- individuals at baseline, 30 (18%) converted to MCI. In comparison, 5 (63%) of the 8 GZ individuals and 11 (55%) of the 20 Aβ+ individuals converted to MCI. A Cox Proportional Hazard model was applied to assess the risk of MCI conversion with baseline CL as the main predictor and age and years of education as significant risk factors (Fig. 4). Having higher amyloid load at baseline presented an increased hazard for MCI conversion (GZ and Aβ+ groups, HR 3.44 and 3.04, respectively), as well as being older (HR 1.09). On the other hand, higher education slightly lowered the risk of MCI conversion (HR 0.94).

### Longitudinal assessment

#### Centiloid classification progression

118 participants underwent all three PET scans (Years 0, 2 and 5), 51 had two scans ~ 2 years apart (Years 0 and 2), 27 had two scans ~ 3 years apart (Years 2 and 5) and one participant had two scans ~ 5 years apart (Years 0 and 5). On average, PET scan follow-up time was 4.2 ± 1.5 years. The CL classification changes are in Table 3.

**Table 3:** Baseline and final amyloid status classification based on CL values from [^18^F]florbetaben PET scans

		Final CL classification		
		Aβ-	Grey Zone	Aβ+
Baseline CL classification	Aβ-	153 (90.53%)	9 (5.33%)	7 (4.14%)
	Grey Zone	-	2 (25%)	6 (75%)
	Aβ+	-	-	20 (100%)

**Fig. 5:**
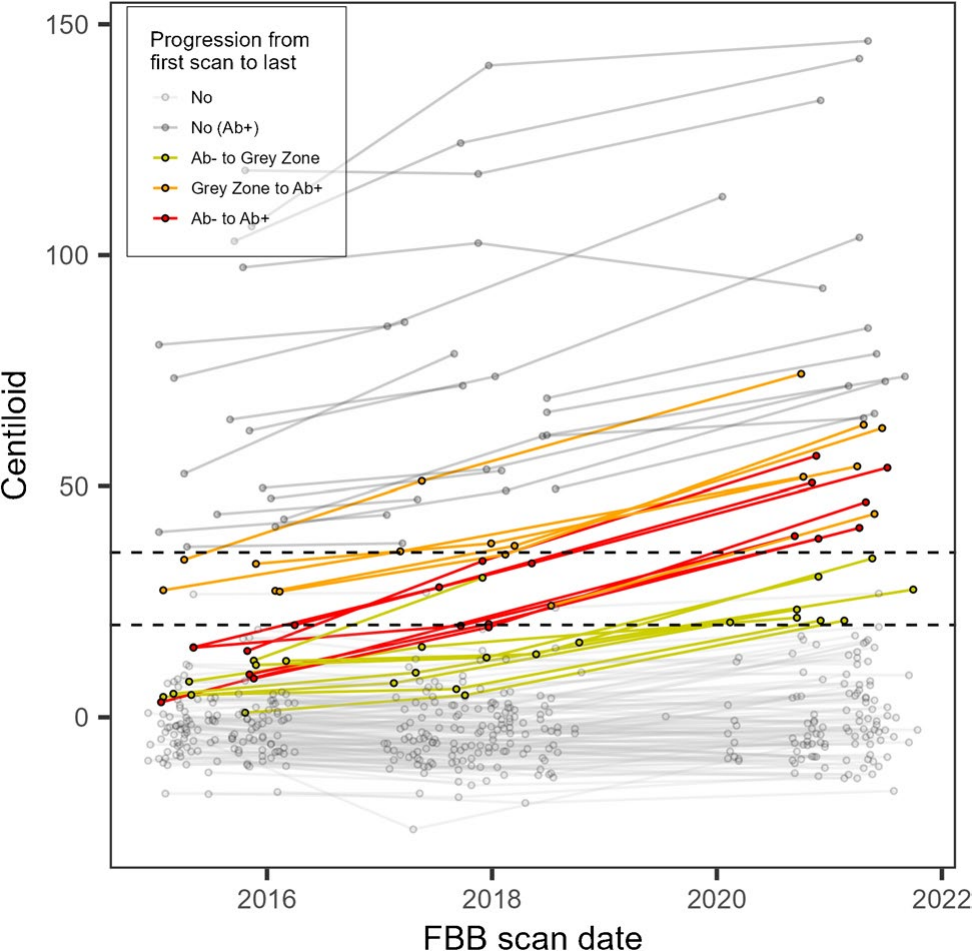
Longitudinal change in CL. Individuals that changed classification between first and last scan are highlighted in yellow, orange or red depending on their progression. Horizontal dashed lines show the cut-off for Aβ- (CL ≤ 20) and Aβ+ (CL > 35.7) scans. Individuals that were Aβ+ at baseline are displayed in slightly darker grey

The CL for each scan is presented in Fig. 5. No CL classification change is observed for most Aβ- participants, with only 5% advancing to the GZ and 4% to Aβ+. From the 8 individuals in the GZ at enrolment, 6 (75%) progressed to Aβ+. No individuals enrolled with Aβ+ classification changed amyloid status (and most showed continued accumulation of amyloid).

#### Centiloid annualised rate of change

**Fig. 6:**
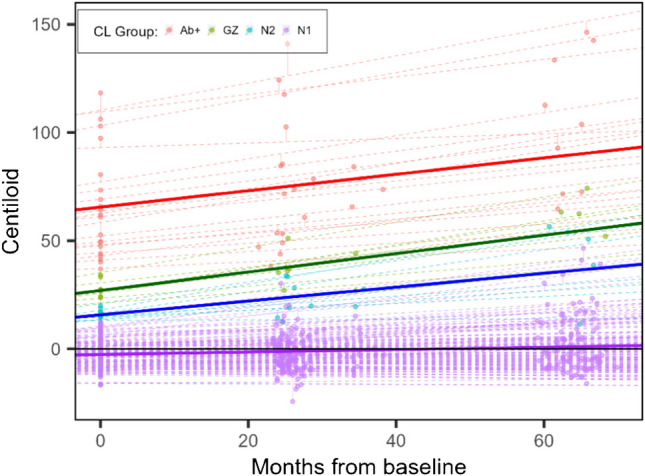
Visualisation of the Linear Mixed Model results (lines) with the measured CL values (dots). The dashed lines correspond to individual-level results and the full lines are group-level results

With the interest of a detailed investigation of Aβ- individuals, this group was split into N1 (CL ≤ 13.5, low/no Aβ) and N2 (13.5 < CL ≤ 20, close to GZ) so that the amyloid accumulation of these populations could be independently evaluated. The LMM estimates for group intercept (baseline CL) and slope (CL ARC) are shown in Table 4. Despite N1 and N2 groups being both Aβ-, they present a substantially different ARC (0.68 CL/year and 3.84 CL/year, respectively). Meanwhile, the Aβ+ group has a lower ARC (4.55 CL/year) than the GZ group (5.10 CL/year). This can also be seen in Fig. 6, where intercepts and slopes are shown at group-level and individually (fixed and random effects of the LMM, respectively).

**Table 4:** Linear Mixed Model results and number of amyloid accumulators in each group defined by baseline CL

CL Group	Group cut-off	*N*	Group estimated baseline CL*	Group ARC* (CL/year)	Number of Aβ accumulators
Aβ- N1	CL ≤ 13.5	160	− 2.59 (− 4.10, − 1.07)	0.68 (0.36, 1.00)	19% (*n* = 31)
Aβ- N2	13.5 < CL ≤ 20	9	15.73 (9.15, 22.31)	3.84 (2.44, 5.23)	89% (*n* = 8)
GZ	20 < CL ≤ 35.7	8	27.02 (20.07, 33.97)	5.10 (3.67, 6.54)	100% (*n* = 8)
Aβ+	35.7 < CL	20	65.66 (60.99, 70.10)	4.55 (3.54, 5.55)	95% (*n* = 19)

*The intercept (estimated baseline CL) and slope (ARC) are significantly different than zero (p < 0.001) for all groups. Aβ accumulators are defined below

**Fig. 7:**
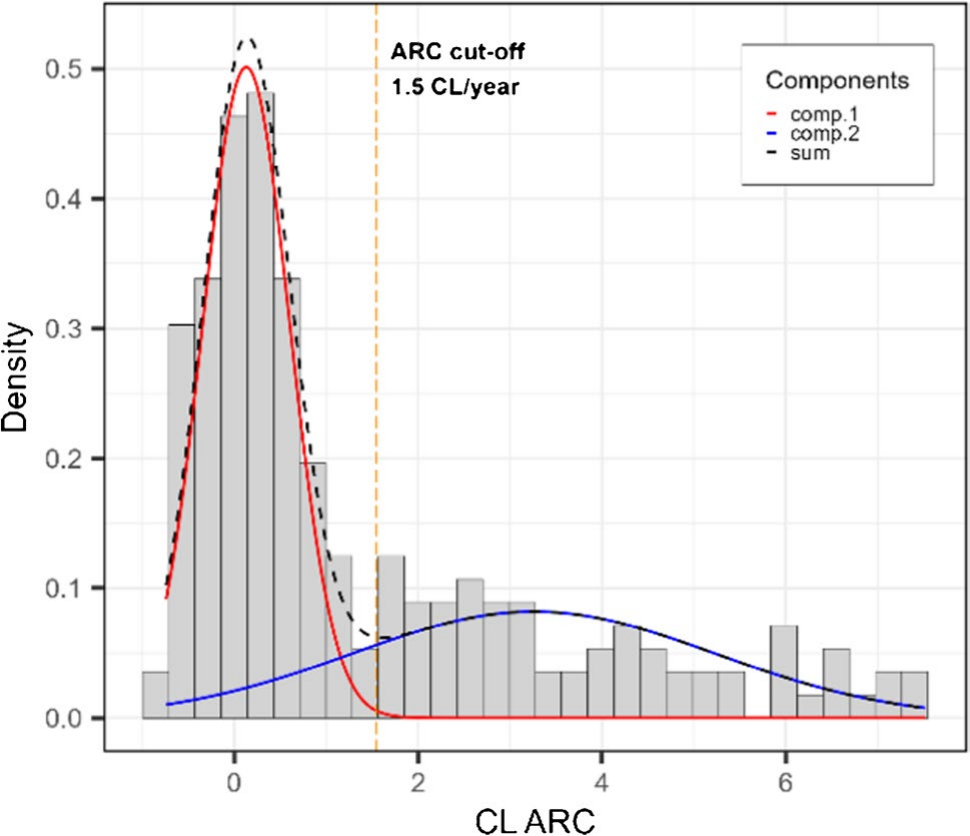
Histogram of the CL ARC for the whole cohort fitted with a 2-curve GMM

The ARC distribution is shown in Fig. 7. Given the non-normality of this distribution, a two-curve GMM was applied to the data and yielded a mean and standard deviation of 3.26 ± 1.97 CL/year for the curve with higher mean and 0.13 ± 0.47 CL/year for the curve with the lower mean. A cut-off at 3 standard deviations above the mean of the lowest curve (red line in Fig. 7), defines an individual with substantial amyloid accumulation at a threshold of ARC > 1.5 CL/year. In total, 66/197 individuals are amyloid accumulators (31/160 N1, 8/9 N2, 8/8 GZ, and 19/20 Aβ+; Table 4).

**Fig. 8:**
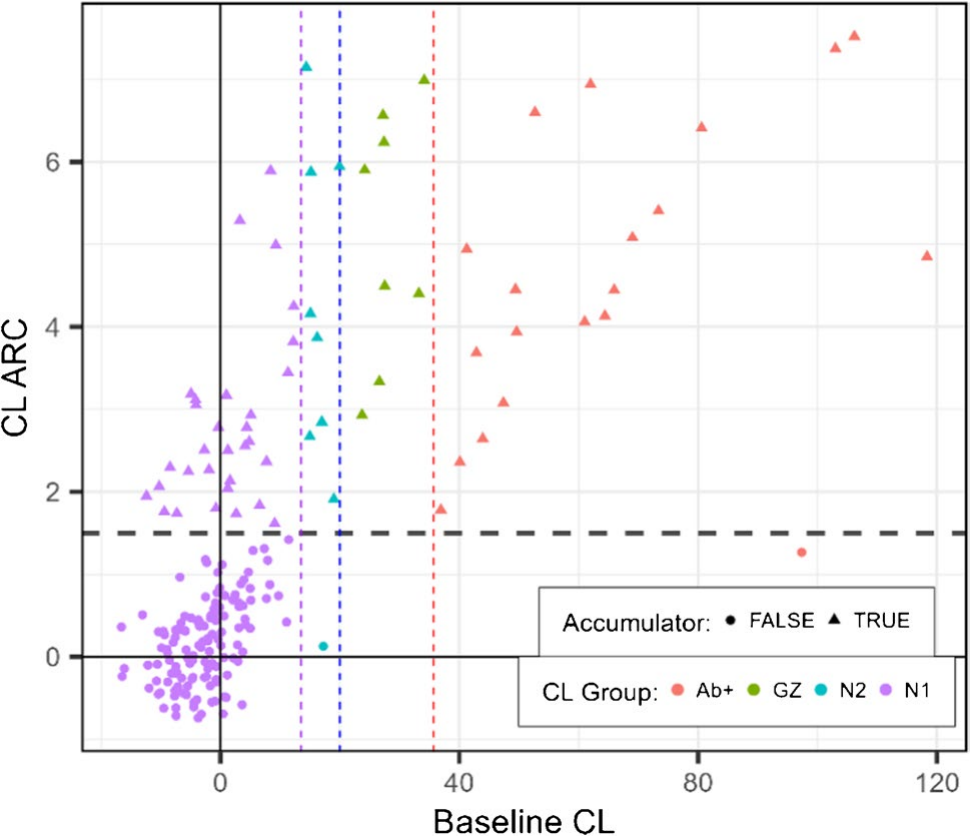
Centiloid annualised rate of accumulation (CL ARC) as function of measured baseline CL. Colours indicate amyloid level groups and dashed vertical lines indicate thresholds for grouping at CL below 13.5 (purple), below 20 (blue), below 35.7 (green) and above 35.7 (red). Dashed horizontal line shows the cut-off for CL ARC classification of individuals with Aβ accumulation. Those considered accumulators are indicated by the triangular symbol

In Fig. 8, CL ARC is displayed as a function of baseline CL. Most N1 individuals have low ARC, however some display surprisingly high ARC that are close to Aβ+ individuals’ ARC. Meanwhile, most in the N2 and GZ groups show substantial CL annual increase. Nearly all Aβ+ individuals have CL ARC in the range of the GZ group, however 4 Aβ+ individuals have ARC lower than the GZ participant with the lowest ARC. Notably, one Aβ + individual has particularly high baseline CL (97.3 CL) and low ARC (1.27 CL/year).

### Spatial pattern in parametric images of N1 individuals

**Fig. 9:**
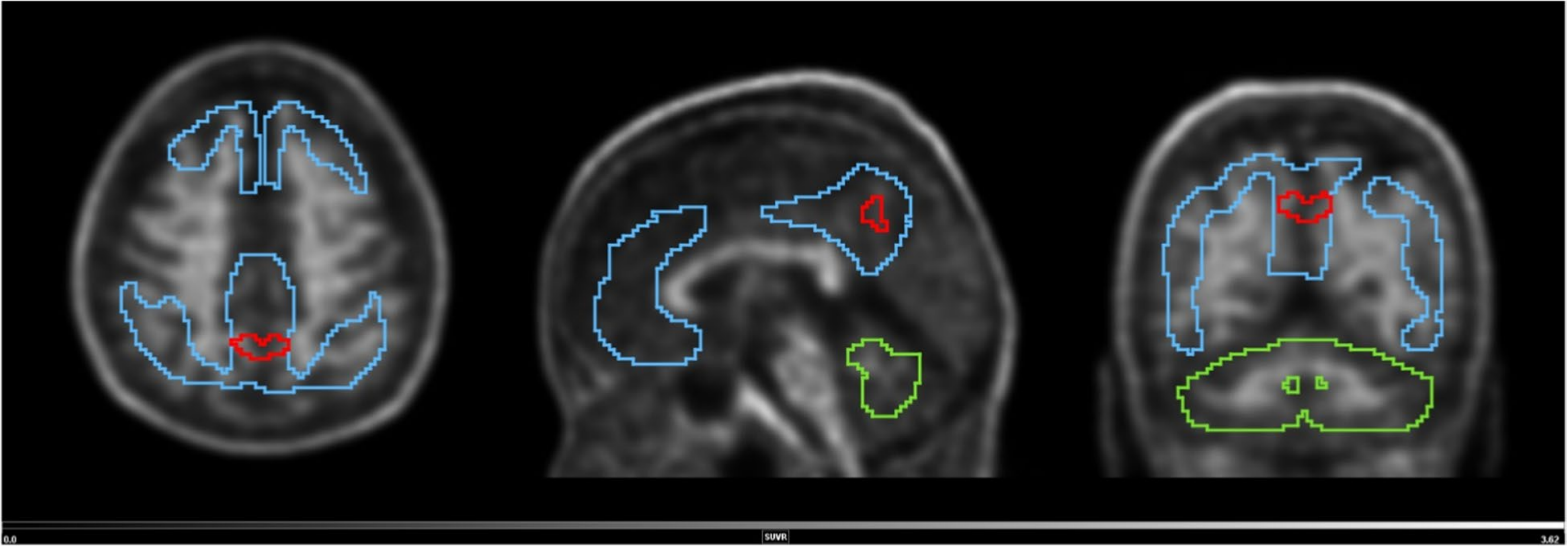
Volume with higher baseline signal for N1 accumulators and N1 non-accumulators of amyloid (red line) overlayed on the scan of an N1 accumulator (MNI space). Grey scale shows SUVR range. Blue line shows the Centiloid target region and the green line shows the reference region (whole cerebellum)

To better understand the sub-threshold N1 group, the baseline scans of N1 Aβ accumulators and N1 with stable amyloid were compared. The voxel-wise SPM analysis detected a small region within the precuneus that showed higher baseline SUVR in N1 Aβ accumulators compared to N1 non-accumulators (*p* < 0.001). This region is roughly symmetrical in relation to the longitudinal fissure and near the interface between the precuneus and the cingulum (Fig. 9), has a total volume of 1.864 mL (233 voxels), a mean SUVR of 1.00 ± 0.10 for accumulators and 0.87 ± 0.07 for non-accumulators, and is within the standard Centiloid target volume of interest. The baseline CL for the N1 sub-groups were − 2.8 ± 5.8 and 1.1 ± 6.9 for non-accumulators and accumulators, respectively (*p* < 0.01, two-sample t-test). In a bilateral regional analysis, the precuneus grey matter mean baseline SUVR was 0.92 ± 0.04 for non-accumulators and 0.95 ± 0.06 for accumulators (*p* = 0.0098, Welch two sample t-test). After correction for multiple comparisons, the only AAL region to show differences between N1 sub-groups was the posterior cingulum (baseline SUVR 1.10 ± 0.05 for non-accumulators and 1.14 ± 0.07 for accumulators; two sample t-test *p* = 0.038).

## Discussion

In this study we analysed longitudinal amyloid accumulation and cognitive decline as a function of baseline CL values assessed by [^18^F]florbetaben amyloid brain PET in a cohort of 197 individuals with SCD from the FACEHBI cohort followed-up for up to 5 years.

Our data showed significant differences in longitudinal amyloid accumulation depending on the baseline CL. Only around 10% of individuals who were Aβ- at baseline developed emerging amyloid pathology (GZ) or pathological levels of amyloid burden (Aβ+) in the 2–5 years after the initial scan. The GZ group showed the fastest amyloid accumulation, while the Aβ+ group presented a slight slowdown. Despite the overall low Aβ accumulation rate of N1 group, 19% of those individuals presented a higher baseline Aβ PET signal in a small part of the precuneus when compared to N1 non-accumulators. This finding could support very early indication of SCD individuals being at increased risk of future cognitive decline, especially because higher amyloid loads increase the chance of conversion to MCI, particularly in those who are older and/or have lesser education.

Most study participants were classified at baseline as Aβ- due to low amyloid level in the brain (*n* = 169/197 (86%), CL ≤ 20). Nevertheless, despite normal cognitive scores, several individuals presented high amyloid burden (*n* = 20/197 (10%), CL > 35.7) and some showed emerging amyloid load (*n* = 8/197 (4%), 20 < CL ≤ 35.7). This is in line with data from previous independent studies of cognitive unimpaired cohorts [2, 31, 44, 45], however our study has a lower rate of Aβ+ participants than previous publications, which could be attributed to differences in recruitment strategies.

An increased risk of progression to MCI was observed for individuals with higher amyloid load at baseline (GZ and Aβ+ groups), who were older and with fewer years of education. Interestingly, the APOE-ε4 carriership did not play a role in the risk of cognitive decline in the present analysis despite a trend for significance (*p* = 0.06). A key implication of identifying these risk factors for future cognitive decline is their potential to inform enrolment criteria for trials of disease-modifying drugs and to assist in selecting patients for approved treatments. However, it remains uncertain if the cognitive decline observed in all participants who converted to MCI is related to AD, as MCI is a clinical diagnosis with several potential underlying causes. With fluid biomarkers becoming more relevant in AD research, it is important to highlight that the FACEHBI cohort has been assessed with some of these biomarkers. Lower plasma Aβ42/Aβ40 ratio values (quantified by a mass-spectrometry assay) were associated with poorer episodic memory, brain atrophy, and amyloid PET burden (CL > 13.5), and were able to predict clinical conversion from SCD to MCI at 2-year follow-up [32]. Furthermore, the FACEHBI cohort was evaluated by retinal thickness from Optical Coherence Tomography (which was associated to Aβ PET positivity but not with conversion to MCI over 24 months [28]) and with white matter hyperintensities from MRI (which were associated with poorer episodic memory performance [30]). Studies on other SCD cohorts showed that individuals with baseline CL < 50 had stable MMSE scores while a baseline CL > 75 was related to MMSE decrease [31]; that an Aβ+ scan increased progression from MCI to AD dementia [17]; that higher Aβ burden was related to increased rate of memory and executive function decline [16, 27]; that some SCD cognitive scores can increase the likelihood of subsequent Aβ PET positivity [29]; and that years of education was negatively associated with CL values [33]. The present analysis was in line with these studies and showed the capability of amyloid PET to identify the risk of cognitive decline in the pre-clinical phase of possible AD even in a population with very low amyloid burden.

Regarding the longitudinal progression of Aβ burden in the brain, only 16/169 individuals who were initially Aβ- had sufficiently high amyloid load in their final scan to change CL classification to either GZ (*n* = 9; 5.3%) or Aβ + (*n* = 7; 4,1%). In contrast, most of the baseline GZ participants progressed to Aβ+ (*n* = 6; 75%). However, to define an individual as an *Aβ accumulator*, a threshold of 1.5 CL/year was found based on the LMM and GMM analyses. While other studies have assessed amyloid accumulation, only Bollack and colleagues [21] established thresholds to determine who could be classified as Aβ accumulator using data from two cohorts: AMYPAD-PNHS [4] and Insight46 [46]. Two methods were employed to define the accumulation threshold in Bollack’s study: at the 95 th percentile of the ARC distribution in a reference population (3.7 CL/year for AMYPAD-PNHS and 3.0 CL/year for Insight46), and at the 95 th percentile of the lowest component of a 2-curve GMM applied to the ARC distribution (3.2 CL/year for AMYPAD-PNHS and 2.2 CL/year for Insight46). These values are higher than the one in the present study mostly due to the different thresholds for defining the Aβ-, GZ and Aβ+ groups, ARC calculation method and likely due to the inclusion criteria in each cohort. With Bollack’s GMM cut-off methods applied to the FACEHBI cohort (which was part of the AMYPAD-PHNS), the following values were found: 3.8 CL/year (95 th percentile of the ARC of the Aβ- group) and 0.91 CL/year (95% quantile of the lowest GMM curve). The former cutoff is in line with Bollack’s work however the latter is substantially lower than what was observed by their work. The higher value resulting from Bollack’s work comes from the GMM components (Fig. 1 of [21]) overlapping considerably more than ours (Fig. 7), a consequence of more individuals with negative ARC and a gradual decrease of the ARC distribution from 0 CL/year to high values. The LMM approach with multiple scans should be more reliable than an average change based on the difference between two scans and assessed by previous studies. However, the test–retest variability of [^18^F]florbetaben PET scans (~ 3–6%) [47–49] does put a constraint on how much an increase of 1.5 CL per year is measurable, especially if: injected doses vary significantly, scanning protocol changes, or the follow-up scan is performed on another machine [50–52]. Therefore, if Aβ PET follow-ups are envisioned for future clinical and treatment monitoring, intervals longer than one year between PET scans may yield a more accurate measurement of amyloid accumulation compared to annual scans. Of note, other studies assessed regional signal of Aβ and found that accumulation rates may vary for different brain regions [16, 45, 53], however this is outside of the scope of the present work.

Regarding group-based ARC, the baseline GZ group had the highest ARC (5.10 CL/year), and all individuals showed Aβ accumulation. Meanwhile, the Aβ+ group showed an ARC of 4.55 CL/year, and 95% of individuals were Aβ accumulators. This slow-down of the accumulation rate in Aβ+ individuals compared to the GZ group is consistent with the sigmoidal model of amyloid deposition in which biomarker levels reach a plateau over time [11] and similar trends have been reported previously [2, 17, 21, 27, 44, 54]. In this study we observed that the Aβ levels can be abnormal at baseline, present a rapid increase (even for subjects with very low Aβ burden like the N1 group), and start approaching plateau levels few years after self-reporting of cognitive decline. Such understanding could impact the patient selection for anti-amyloid treatment trials and patient management [23, 55].

To better understand the trajectory of individuals below the Aβ- threshold, two sub-groups were studied: N1 (CL around 0) and N2 (CL close to the GZ threshold). The N1 sub-group presented markedly low ARC (0.68 CL/year) while N2 had substantial Aβ accumulation (3.84 CL/year), demonstrating that these sub-groups indeed represent two populations with different longitudinal trajectories regarding Aβ deposition. The N2 group presented a similar behaviour to the GZ group, therefore supporting the arguments for an extension of the GZ CL range. Despite very low baseline Aβ burden 31 individuals in the N1 group were Aβ accumulators and showed a small region within the precuneus with significant higher baseline PET signal than the remaining 129 N1 individuals. The precuneus is known to be one of the cortical regions with early amyloid accumulation in AD [2, 16], is relevant for amyloid staging models [56, 57], and its focal signal can lead to positive visual PET reads on images with low CL values [31]. Therefore, the presented results show that even at a very low Aβ burden this pattern could be recognised, highlighting the power of amyloid PET quantification in detecting very early signs of amyloid pathology in the brain and supporting existing evidence of early brain changes due to Aβ burden. The identified region is within the CL target region, which explains why the Centiloid method was, to a certain degree, capable of identifying differences between the N1 accumulators and non-accumulators. However, this should be interpreted with caution as it cannot be assumed that a higher signal in this region will inevitably accumulate Aβ and lead to cognitive decline.

A clear limitation of this study is the relatively small sample size, especially for the GZ and Aβ+ groups, given the heterogeneity of SCD individuals. As a single centre study, image quality and processing harmonisation is straightforward, but generalisability of results might be limited. Furthermore, there is no definitive consensus in the PET community of which CL values should be used to define Aβ+ and GZ groups [58]. Therefore, despite the robustness of the CL method [22], it certainly makes study comparisons more difficult.

## Conclusion

This study demonstrated the ability of Aβ PET to detect early amyloid deposition in the brain of individuals with subjective cognitive decline, including the identification of rapid amyloid accumulation in otherwise normal brains. A distinct region within the precuneus was found to exhibit baseline Aβ loads associated with an increased risk of subsequent widespread amyloid accumulation in individuals without initial amyloid deposition. Higher amyloid levels, older age and lower education were related to an increased risk of conversion to mild cognitive impairment. These findings suggest a promising approach for detecting pre-clinical Alzheimer’s disease within a population experiencing subjective cognitive decline, which may aid in the appropriate selection of individuals for effective interventions.

## Data Availability

The data supporting this study is available from MM on reasonable request. This data is also part of the AMYPAD, which can be retrieved from the Alzheimer’s Disease Data Initiative (ADDI, https://www.alzheimersdata.org/).
